# Socioeconomic and Psychosocial Mechanisms Underlying Racial and Ethnic Disparities in Cognition Among Older Adults

**DOI:** 10.1093/geroni/igaa057.2829

**Published:** 2020-12-16

**Authors:** Laura Zahodne, Neika Sharifian, A Zarina Kraal, Emily Morris, Afsara Zaheed, Ketlyne Sol, Jennifer Manly, Adam Brickman

**Affiliations:** 1 University of Michigan, Ann Arbor, Michigan, United States; 2 Columbia University, New York, New York, United States

## Abstract

Racial/ethnic disparities in cognitive aging are only partly attributable to socioeconomic indicators. Emerging literature highlights psychosocial factors, such as related constructs of discrimination and perceived control. Using data from 1,463 older adults (51% Hispanic, 27% non-Hispanic Black, 22% non-Hispanic White) in the Washington Heights-Inwood Columbia Aging Project, cross-sectional mediation models quantified separate indirect effects of Black race and Hispanic ethnicity on global cognitive composite scores. Socioeconomic status explained approximately 50% of Black-White and Hispanic-White disparities in cognition. Perceived control explained an additional 5-8%. Discrimination was not associated with cognition. Significant racial/ethnic disparities remained after accounting for the included socioeconomic and psychosocial factors, indicating that future studies should consider additional potential mediators. Lower perceived control, which likely reflects chronic exposure to interpersonal and institutional marginalization, may be a particularly salient psychosocial risk factor for poorer cognitive aging among certain racial/ethnic minority groups.

